# Direct Polypropylene and Polyethylene Liquefaction in CO_2_ and N_2_ Atmospheres Using MgO Light and CaO as Catalysts

**DOI:** 10.3390/ma15030844

**Published:** 2022-01-22

**Authors:** José Miguel Hidalgo Herrador, Martyna Murat, Zdeněk Tišler, Jakub Frątczak, Héctor de Paz Carmona

**Affiliations:** ORLEN UniCRE a.s., Revoluční 1521/84, 400 01 Ústí nad Labem, Czech Republic; martyna.m.murat@gmail.com (M.M.); zdenek.tisler@orlenunicre.cz (Z.T.); jakub.fratczak@orlenunicre.cz (J.F.); hector.carmona@orlenunicre.cz (H.d.P.C.)

**Keywords:** polyethylene, polypropylene, direct liquefaction, MgO, CaO, plastic, waste

## Abstract

The polyolefin to lighter molecules reaction reduces the waste-plastic residues to produce fuels and valuable chemicals. Commercial MgO light and CaO were used as catalysts for the direct polyethylene and polypropylene liquefaction in N_2_ or CO_2_ atmospheres. The products were analyzed (ATR-FTIR, GC-FID/TCD, GC-FID, density, refractive index). The use of MgO light and CaO improved the conversion of propylene and ethylene to liquid products. In addition, low gaseous and solid products yields were obtained. A good production of organic liquids in the gasoline, diesel and kerosene boiling range was obtained. The use of CO_2_, in some cases, led to a higher conversion into liquids compared with the reactions performed in the N_2_ atmosphere. In addition, the use of the CO_2_ atmosphere led to a higher content of products with a boiling range in the diesel and kerosene ranges.

## 1. Introduction

The increasing prices and demand, the threat of global warming and the scarcity of fossil fuels and chemicals are increasing the global need for the production of environmentally friendly and renewable fuels and chemicals. In addition, the processing of plastics is a big problem nowadays. Plastic residues are long-term degradable waste that cause marine litter or land sediments, affecting the environment [1,2,3]. Plastic residues can be collected and converted to fuels or chemicals to decrease the waste residue problems [3].

In general, the existing methods used to reduce plastic waste accumulation are not effective. Incineration with the subsequent potential toxic gas production and physical recycling, such as clean PET waste, is limited by repeated cycles and gradual degradation [4]. Another procedure to produce valuable products from waste plastic is the pyrolysis reaction. This reaction is a thermal degradation of long-chain molecules to produce short-chain molecules. It has a long history, from the pyrolysis of wood to produce coal to the pyrolysis of plastics in the last 30 years [5]. In terms of using waste plastic, there is the advantage of waste plastic having a lower oxygen content and higher carbon contents compared with the biomass oxygen and carbon contents. The pyrolysis of polymers can produce organic products with up to 80 wt% liquid [5,6,7,8].

Direct liquefaction is another type of reaction that can be used to obtain oil liquids from many raw materials (biomass, plastics, coal, among others). It is a useful reaction to obtain oil liquids. It consists of the thermal degradation of the feedstock mixed with a solvent. This solvent helps to increase the content of liquid product [8,9,10]. The liquid products obtained by low-temperature pyrolysis or the direct liquefaction process need to be then distilled, purified and upgraded in order to be employed as transport or chemicals in the industry. Even though direct liquefaction could produce fuels that are an alternative to petroleum, an additional purification process is often required depending on the market and industry needs [11].

Many catalysts have been described for the pyrolysis of polyolefins such as zeolites (ZSM-5, Y-zeolite, natural zeolites, red mud, among others) [12]. The MgO was proven to be a valuable and cost-efficient catalyst for the pyrolysis of plastics [13]. The CaO is another cost-effective catalyst that could be tested for the direct liquefaction tests, especially because of its catalytic activity in the pyrolysis of plastics [14]. Therefore, MgO and CaO could be good candidates for being tested in the direct liquefaction reaction of plastics. Concretely, MgO commercial can be found as MgO light and MgO heavy [15] when obtained from the natural magnesite source. Porous MgO can be obtained by calcinating the mineral at a lower temperature (700–1000 °C) and obtaining the solid with the most significant specific surface area. This type of porous MgO is obtained commercially and is called MgO light. So, these materials can be considered cost-effective potential catalysts for the direct liquefaction of polyethylene (PE) and polypropylene (PP). No publications about the direct liquefaction of PE or PP using CaO or MgO were found (even when CO_2_ atmosphere was included), so research using these solids for the polyolefins could provide information about the direct plastic liquefaction.

In this work, MgO light (a commercial porous material) and CaO standard are readily-available and low-cost commercial solids-catalysts that were used for the direct PP and PE liquefaction in N_2_ and CO_2_ atmospheres. The aim was to obtain shorter-chain molecules from the longer-chain molecules polymers, PE and PP, which could then be used as liquid fuels. Depending on the use of MgO and CaO (in N_2_ or CO_2_), the amount of liquid and their different boiling range fractions changed.

## 2. Materials and Methods

### 2.1. Materials

PE and PP homopolymers (both commercial raw materials) “HDPE LITEN” and “PP MOSTEN” supplied by ORLEN Unipetrol were used for the reaction [16,17]. Tetralin (Honeywell, Honeywell, Charlotte, NC, USA, ≥97%) was used as solvent. Commercial MgO light G.R. (98%) and CaO (Lach-Ner Ltd., Neratovice, Czech Republic) were used as catalysts.

### 2.2. Tests

Twelve tests were carried out including tests without a catalyst. The reaction tests were performed at 420 °C, 1 MPa (N_2_ or CO_2_), TOS = 1 h, 50 g of tetralin, 10 g of polymer and 2 g of catalyst (MgO or CaO). The tests’ names indicated whether the feedstock was PE or PP, whether the atmosphere was N_2_ (N2) or CO_2_ (CO2) and whether the catalyst was CaO or MgO, including the name of the catalysts (CaO and MgO). Tests PE-N2, PP-N2, PE-CO2 and PP-CO2 were carried out without the catalyst, with PE or PP in N_2_ or CO_2_. For example, PP-MgO-N2 was performed using PP and MgO in the N_2_ atmosphere.

An autoclave 4575/76 with a controller “4848B” (Parr Instruments Company) was utilized for all the tests. The reactor was flushed with N_2_ or CO_2_ to remove air residues and pressurized to 10 bar with N_2_ or CO_2_. The temperature was then increased from room temperature (25 °C) to 420 °C with an average of 8.3 °C min^−1^. Then, the reactor was at 420 °C for 1 h. The next step was the external cooling of the air flow (average cooling rate of 4.5 °C min^−1^) to ambient temperature and stabilization for 1 h. After finishing the previous process, the gas was sampled and the autoclave depressurized. After that, the autoclave was opened, and the liquids and solids were collected for analysis.

### 2.3. Mass Balance

When each test finished, the reactor was weighed for liquids and solids. Thus, the amount of solids + liquids was calculated. By working out the difference, the amount of gas was also calculated.

The mass balance was calculated for liquids and solids by the weight of the total sediment and liquids filtrated (first cold filtration). Then, the liquids were centrifuged in vials of 40 mL at 2600 rpm for 30 min, obtaining a clean liquid on the upper side and small amounts of semisolids (solid + non miscible liquid mixtures) into the bottom. For final mass balance results (solid + liquid + gas), 2 g were subtracted from the total solids (2 g of initial catalyst were used). The products were divided into gases, organic liquids (non-soluble in water), aqueous liquids (water soluble) and solids.

After calculating the solids + liquids + gases mass balance, the reactor contained some solids strongly adhered to the walls. These solids could only be cleaned using solvents (xylene, cyclohexane, acetone, isopropanol), high temperatures (up to 495 °C in air flow), mechanical methods using a drill or a combination of the three. They were considered as losses (solid residues).

### 2.4. Analytical Methods

The gaseous products were analyzed by gas chromatography using a Refinery Gas Analysis (RGA) (GC model 7890A, Agilent Technologies, Santa Clara, CA, USA). This method was configured to analyze refinery gas up to C6 hydrocarbons including H_2_, O_2_, N_2_, CO, CO_2_, hydrogen sulfide (H_2_S) and carbonyl (COS) sulfides, respectively.

The liquids (organic phase) were analyzed by simulated distillation (SIMDIS) by using the ASTM D7169 method [18]. The refractive index at 20 °C was obtained by using an RFM 970 automatic refractometer (Bellingham + Stanley, Tunbridge Wells, Kent, UK) according to ASTM D 1218-02 [19].

The density was determined by using a Specific Gravity Meter KEM DA-645 (Mettler Toledo, Giessen, Germany) according to the standard ČSN EN ISO 12185 [20].

Thermal analyses (TGA) were performed using a TA Instruments Waters LLC instrument (Mettler Toledo, Giessen, Germany) from 50 to 900 °C, using a heating rate of 10 °C min^−1^ under both nitrogen and oxygen atmospheres, with a 50 mL/min flow.

An ICP-OES/Agilent 725 instrument (Agilent Technologies, Santa Clara, CA, USA) was used to analyze the elemental composition of the feedstocks [21].

The attenuated total reflectance technique (ATR) was used for the FT-IR analyses. All samples were previously dried in a glass-cell at 110 °C under vacuum (16 h) using an instrument called the Nicolet iS10-Thermo Scientific ((Thermo Scientific, Waltham, MA, USA) (crystal diamond; number of scans = 64; resolution 4 cm^−1^).

## 3. Results and Discussion

### 3.1. Feedstocks Analyses

**Figure 1:**
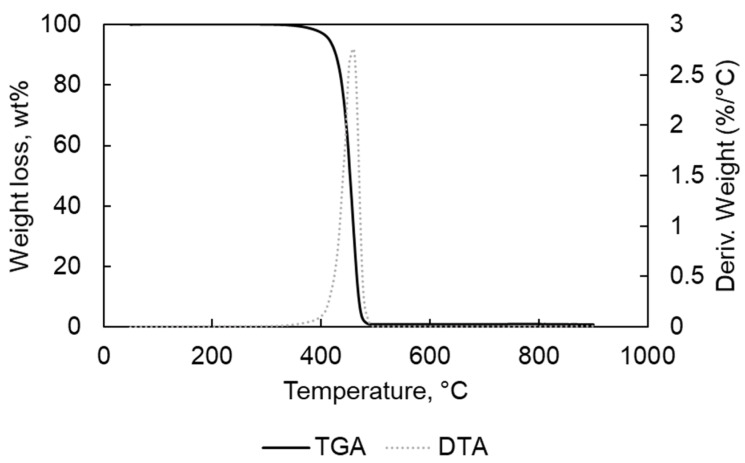
TGA and DTA analysis for PP in N_2_ atmosphere.

**Figure 2:**
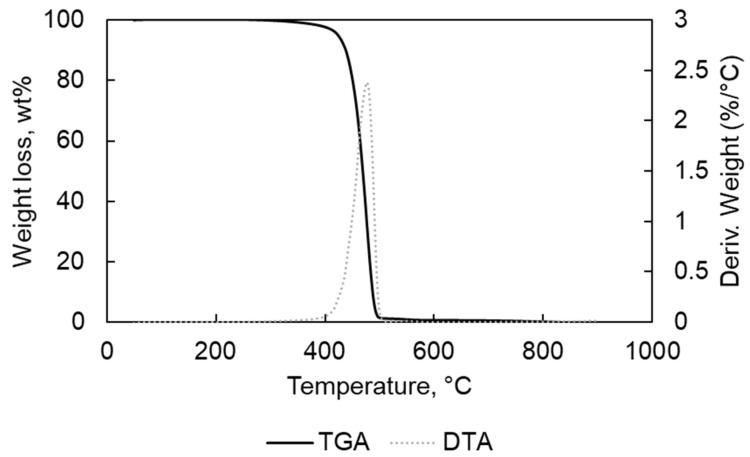
TGA and DTA for PE in N_2_ atmosphere.

**Figure 3:**
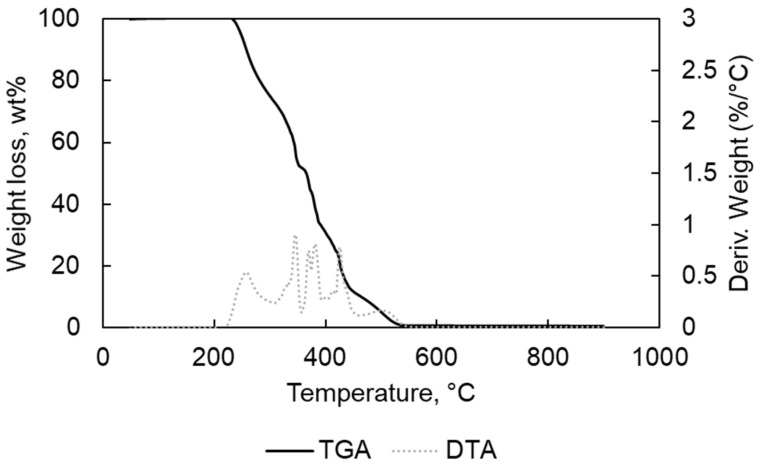
TGA and DTA for PE in O_2_ atmophere.

**Figure 4:**
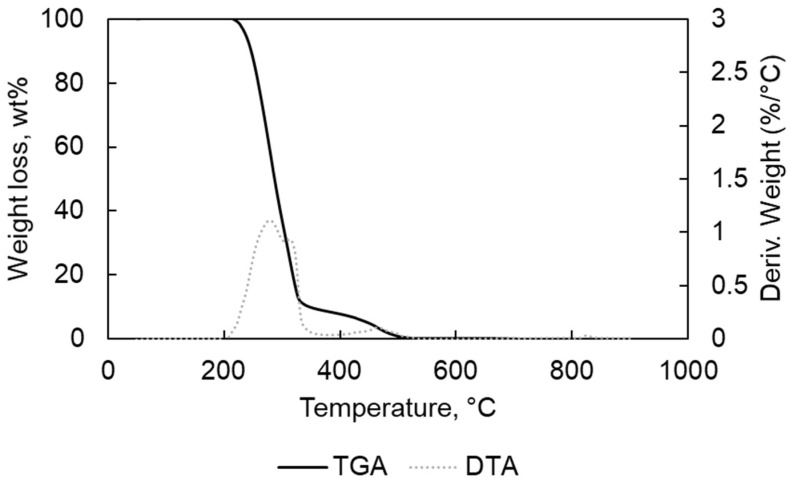
TGA and DTA for PP in O_2_ atmophere.

In Table 1, the results of the elemental analyses for PP and PE are written; they are free of halogens and low metal contents as confirmed by the TGA-DTA analyses results (Figure 1, Figure 2, Figure 3 and Figure 4).

**Figure 5:**
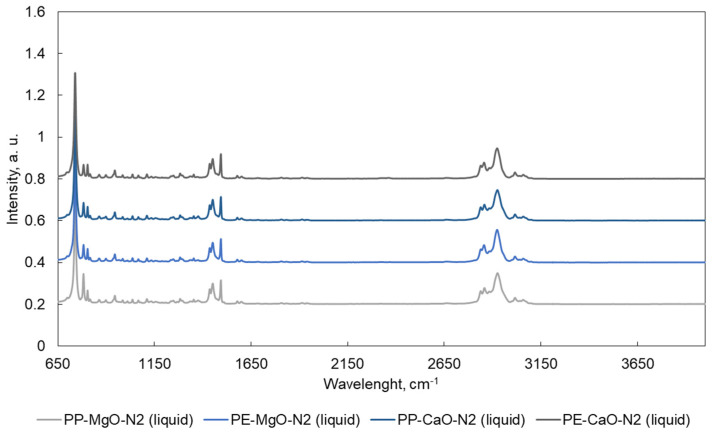
ATR spectra for the liquid products obtained after the tests performed in N_2_. Tests were performed at 420 °C, 10 MPa (N_2_ or CO_2_), TOS = 1 h, 50 g of tetralin, 10 g of polymer and 2 g of catalyst.

The TGA analyses were carried out for each feedstock to determine the optimum temperature for thermal degradation. Figure 1, Figure 2, Figure 3, Figure 4 and Figure 5 present the thermogravimetric (TGA) curves for PE and PP samples. One main loss of weight was found for the analyses carried out in N_2_. The first weight loss started at 315 and 350 °C, finishing at 504 and 495 °C for PE and PP samples, respectively (Figure 1 and Figure 2). In the case of the TGA for PE, a second slight weight loss (<0.3 wt%) was located in the 495–800 °C. The final weight, at the end of the measurement, near to 0 wt% confirmed the low content of ash in PE. In the case of PP, the weight loss was almost 0 wt% at the end of the thermal degradation. However, for the PE, the final residue contained 0.53 wt% of the original starting weight. The only main weight loss could be due to the homogeneous structure decomposition of the polymers PP and PE [22].

For the oxidative TGA decomposition of the two polymers PP and PE (Figure 3 and Figure 4), the steps of decomposition correspond to the presence of a carbon-carbon bond that promotes a random scission mechanism with the increased temperature [22]. The start of the degradation at low temperatures (210–250 °C) could mean that the polymers (PE and PP) are characterized by low molecular weight. The shorter the chains, the more prone they are to thermal and oxidative degradation at lower temperatures [23]. The final stage (8.5 wt% for PE and 9 wt% for PP) could be generated by the final oxidation of the residues [24]. For the samples, TGA under O_2_ have shown more decomposition stages especially for PE compared with TGA analyses under N_2_.

For the PE, the first weight-loss stage was found at 235–282 °C (−19.7 wt%), the second one at 282–352 °C (−33.9 wt%), the third one at 352–409 °C (−22.3 wt%), the fourth at 409–457 °C (−15.1 wt%) and the fifth at 457–544 °C (8.5 wt%). The final residue weight was due to a final ash-residue of 0.44 wt%, which could be mainly metal oxides.

For the PP, the first weight loss was found at 200–292 °C (−60.4 wt%), the second at 292–375 °C (−30.3 wt%), resulting in a residue content of <0.1 wt% at the end of the analyses, taking as reference the initial amount of feedstock.

### 3.2. Product Analyses

The mass balance (gases, semisolids, liquids and losses-solids) results are exposed in Table 2.

The results can be compared in two forms: (i) tests performed in N_2_ and tests in CO_2_ and (ii) reactions carried out without or with different catalysts (MgO and CaO). The tests carried out without using MgO or CaO led to the production of semisolids and gases (no liquids were obtained). N_2_ test results showed a higher yield to liquids when PP was used. In the case of the tests using MgO, the difference was 6.4 wt%. In the case of test performed with CaO, the difference was 26.5 wt%. CO_2_ tests results showed a similar yield of liquids for the tests using PE or PP when MgO was used. However, when CaO was used, the difference in the production of liquids was much higher (35.3 wt%) compared with MgO tests. The lower polyethylene yields could be related to its lower reactivity compared to the polypropylene radical formation during the reaction, taking into account that tertiary radicals (propyl group) are more stable than secondary radicals (ethyl radical group) [25]. The bigger difference in the reactivity of polyethylene and polypropylene when CaO was used could be related to its lower porosity and pore volume. The semisolid contents are related to the unreacted polymer or longer chain polymers, compared with liquid products, which create an emulsion with the tetralin [26]. The polyethylene polymers conversion was lower than the polypropylene molecules, which had a higher content of semisolids found for the blank tests. The quantity of gases was lower than 5 wt% in all cases except for test PE-CaO-CO2 (7.1 wt%). The highest solids content (losses) were found for the two tests, PE-CaO-CO2 and PE-CaO-N2. The highest increment in liquids yields was found from test PE-MgO-N2 to test PE-MgO-CO2. The use of CO_2_ improved the liquid yields when PE and Mg were used (71.8 (N_2_) to 81.9 (CO_2_) wt%).

**Figure 6:**
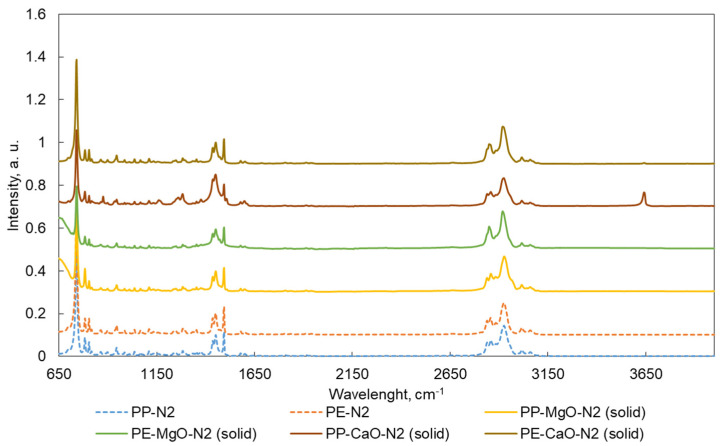
ATR spectra for the solid products (semisolids) obtained after the tests performed in N_2_. Tests were performed at 420 °C, 10 MPa (N_2_ or CO_2_), TOS = 1 h, 50 g of tetralin, 10 g of polymer and 2 g of catalyst.

**Figure 7:**
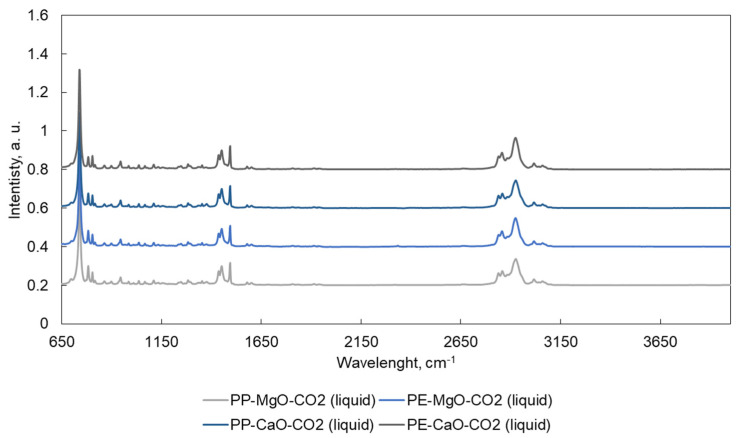
ATR spectra for the liquid products obtained after the tests performed in CO_2_. Tests were performed at 420 °C, 10 MPa (N_2_ or CO_2_), TOS = 1 h, 50 g of tetralin, 10 g of polymer and 2 g of catalyst.

**Figure 8:**
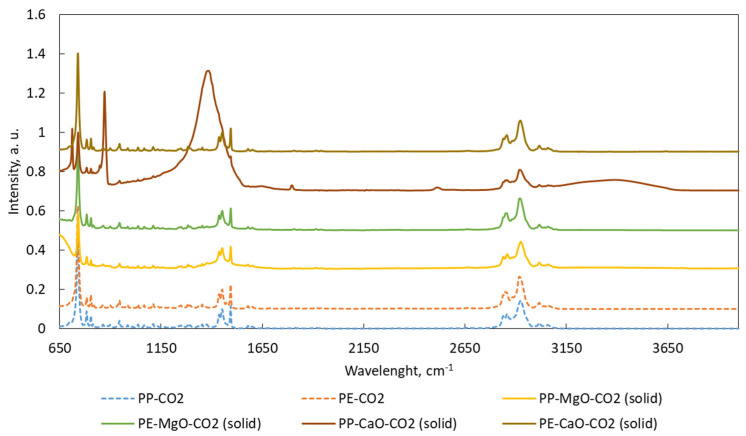
ATR spectra for the solid products obtained after the tests performed in CO_2_. Tests were performed at 420 °C, 10 MPa (N_2_ or CO_2_), TOS = 1 h, 50 g of tetralin, 10 g of polymer and 2 g of catalyst.

ATR-FTIR spectra are shown in Figure 5, Figure 6, Figure 7 and Figure 8. Liquid products (Figure 5 and Figure 7) and semisolid products (Figure 6 and Figure 8) ATR results presented a signal at 727 cm^−1^, which is attributed to the overlapping of the CH_2_ rocking vibration and the out-of-plane vibration of cis-fatty acids [27,28]. The infrared spectra also show a peak at 3009 cm^−1^, attributed to –C=CH (cis double bonds, stretching) [28,29]. The C–H stretch vibrations of the methylene residues were detected at 2928 cm^−1^ (antisymmetric) and 2855 cm^−1^ (symmetric). The bands range at 3100–3000, 1603, 1500 and 900–700 cm^–1^, which is typical for aromatic compounds [30].

ATR-FTIR patterns for the solid products are similar to the results obtained for the liquids. However, the signals located in the 1230–1300 cm^−1^ range (signals related to oxygen bonds C–O) are more intense for the results of the solid sample, especially for the products obtained after the test PP-CaO-N2. In addition, this test presented an additional band at 3640 cm^−1^ (–OH groups connected to CaO) [31,32] indicating contamination of the sample by CaO. The most different results were found in the FTIR results for the solid samples from test PP-CaO-CO2. For these results, the signals at 870 cm^−1^ indicating the presence of Ca–O bonds, the band at 1386 cm^−1^ is the band relative to CaCO_3_ (formed during the reaction in CO_2_ atmosphere) and the band at 1801 cm^−1^ could be related to –OH groups of Ca(OH)_2_. Bands at 2530 cm^−1^ are standardly related to calcite mineral (CaO) and the last different band at 3386 cm^−1^ is related to the -OH groups [31,32,33,34,35]

**Figure 9:**
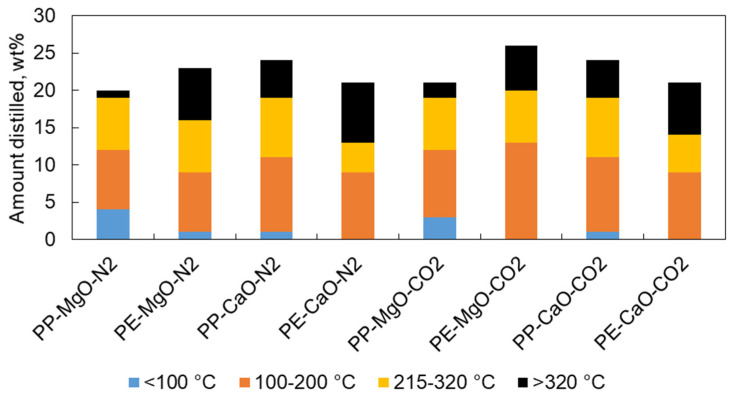
Liquid products distilled amounts at different boiling ranges without taking into account the range of 200–215 °C, attributed mainly to the tetralin solvent boiling range. Tests were performed at 420 °C, 10 MPa (N_2_ or CO_2_), TOS = 1 h, 50 g of tetralin, 10 g of polymer and 2 g of catalyst.

SIMDIS results (Table 3 and Figure 9) show that PP tests led to a significant production of <100 °C boiling range products. PE tests led to a higher percentage of products with boiling ranges >320 °C. The use of CO_2_ instead of N_2_ atmosphere led to similar results except for test PE-MgO-CO2, which increased the production of boiling range 100–200 °C products.

Test PE-MgO-CO2 led to a higher yield of products with a boiling range of 100–200 °C and a total amount of distilled liquid of 26 wt% with respect to the total liquid product. In addition, it has the highest 100–200 °C boiling range fraction (13 wt%). The reason for this high yield of liquids could be due to the significant porosity of the original MgO material which reacts with CO_2_ to produce MgCO_3_, which could present an adequate porosity to adsorb the linear molecules of PE in its surface.

Only a low quantity of gases was obtained, mainly C1–C5 products (Table 4, Table 5 and Table 6). The main gases produced were the C1–C4. No large differences were found between the tests except for the test PE-CaO-N2, in which the gas production was the highest, producing more propylene and C1–C4 products.

The gases wt% were calculated from the total amount of products obtained in wt% subtracting the amount of N_2_ or CO_2_ (reaction carried out using 10 bar (room temperature) of N_2_ or CO_2_).

The refractive index and densities were measured (Table 7). The produces from test PE-CaO-N2 and PE-CaO-CO2 had a higher non-measurable viscosity and density so they were measured at 50 °C.

These results could not be compared directly with other research works because of the different used methodologies [8,9,12,13,36,37,38,39,40,41]. Table 8 shows different results obtained by other researchers and through our work. Two publications using direct liquefaction by using HZSM-5 and without catalysts obtained a higher liquid content in the products compared to our research work when using PE liquefaction [40,41]. However, these experiments were carried out in a tubing reactor [40] or a micro-autoclave [41], using a much lower total amount of feedstock. Other works were performed by the pyrolysis reaction. Compared to the direct liquefaction, this type of reaction led to lower yields to liquids (Table 8) [8,9,12,13,36,37,38,39]. In addition, no direct liquefaction under the CO_2_ atmosphere was found in the literature. The use of CO_2_ implied an increment in the liquid content for the product, especially for test PE-MgO-CO2. This yield increment of liquids was observed and explained in literature but for the pyrolysis reaction for coal [42]. In addition, some more experiments were carried out (Supplementary Materials) with the aim of having more information about the possibilities of this type of reaction.

## 4. Conclusions

Twelve tests were carried out to study the direct liquefaction of model clean polyethylene and polypropylene by using CaO and MgO light commercial solids as catalysts. Almost 100% of the polymer conversion was found to produce mainly liquids as products. The use of catalysts implied that the liquefaction was producing a liquid product, as opposed to the non-catalytic tests producing a semisolid product that could not be distilled. The highest amount of distillable products was found for testing PE-MgO-CO2 with distillable products in the range of 100–200 °C (equivalent to the boiling range of C7–C12 linear paraffins). The use of MgO or CaO as the catalyst and direct liquefaction are suitable potential methods to degrade the polymers. Using a CO_2_ atmosphere implied similar results except for test PE-MgO-CO2, which produces a higher amount of liquids. In addition, the use of CO_2_ implied the production of low amounts of CO.

## Figures and Tables

**Table 1:** Elemental C, H, N, S% analysis and ICP metal analysis of the PP and PE.

Analysis, wt%	PP	PE
N	<0.05	<0.05
S	<0.05	<0.05
C	85.0	85.3
H	14.9	14.6
Al	55.8 × 10^−4^	1.44 × 10^−4^
Ca	24.2 × 10^−4^	8.56 × 10^−4^
Fe	23.3 × 10^−4^	45.5 × 10^−4^
K	11.9 × 10^−4^	2.41 × 10^−4^
Li	<0.2 × 10^−4^	<0.2 × 10^−4^
Mg	33.7 × 10^−4^	0.347 × 10^−4^
Mn	0.238 × 10^−4^	0.284 × 10^−4^
Na	64.5 × 10^−4^	1.20 × 10^−4^
P,	12.9 × 10^−4^	2.93 × 10^−4^
Si, wt%	22.9 × 10^−4^	4.02 × 10^−4^

**Table 2:** Mass balance of the gases, losses, liquid products and semisolids. Tests were performed at 420 °C, 10 MPa (N_2_ or CO_2_), TOS = 1 h, 50 g of tetralin, 10 g of polymer and 2 g of catalyst (MgO or CaO).

Test Abbreviate Name	Liquid	Semisolid	Gas	Losses ^1^
PP-N2	0.0	96.9	1.7	1.4
PE-N2	0.0	97.2	2.8	0.0
PP-MgO-N2	78.6	10.3	4.4	6.7
PE-MgO-N2	71.8	21.5	3.1	3.6
PP-CaO-N2	83.8	6.6	1.3	8.3
PE-CaO-N2	57.3	15.0	3.6	24.1
PP-CO2	0.0	89.0	1.0	10.0
PE-CO2	0.0	96.7	3.0	0.3
PP-MgO-CO2	79.5	7.3	3.6	9.6
PE-MgO-CO2	81.9	6.9	2.1	9.1
PP-CaO-CO2	84.7	10.8	1.2	3.3
PE-CaO-CO2	49.4	14.6	7.1	28.9

^1^ The losses are related to solids adhered to the walls of the reactor (more information in the experimental part (mass balance)).

**Table 3:** SIMDIS for liquid products.

Test Name	<100 °C	100–200 °C	200–215 °C	215–320 °C	>320 °C
PP-MgO-N2, wt%	4	8	80	7	1
PE-MgO-N2, wt%	1	8	77	7	7
PP-CaO-N2, wt%	1	10	76	8	5
PE-CaO-N2, wt%	0	9	79	4	8
PP-MgO-CO2, wt%	3	9	79	7	2
PE-MgO-CO2, wt%	0	13	74	7	6
PP-CaO-CO2, wt%	1	10	76	8	5
PE-CaO-CO2, wt%	0	9	79	5	7

**Table 4:** Gaseous products obtained from non-catalytic tests performed in N_2_ and CO_2_.

	PP-N2	PE-N2	PP-CO2	PE-CO2
C1, wt%	0.12	0.13	0.06	0.12
C2, wt%	0.21	0.21	0.11	0.20
Ethylene, wt%	0.01	0.04	0.00	0.06
Propylene, wt%	0.24	0.23	0.15	0.29
C3, wt%	0.21	0.23	0.12	0.29
C4, wt%	0.37	0.17	0.23	0.26
C5-C6, wt%	0.27	0.19	0.15	0.35
C6+, wt%	0.03	0.06	0.01	0.03
Unknown, wt%	0.08	0.75	0.06	0.43
H_2_, wt%	0.17	0.79	0.06	0.49
CO, wt%	0.00	0.00	0.05	0.49

**Table 5:** Gaseous products obtained from catalytic tests performed in N_2_. The quantity of gases was calculated from the total amount of products obtained in wt%.

	PP-MgO-N2	PE-MgO-N2	PP-CaO-N2	PE-CaO-N2
C1, wt%	0.37	0.24	0.10	0.24
C2, wt%	0.79	0.57	0.19	0.52
Ethylene, wt%	0.02	0.09	0.01	0.06
Propylene, wt%	0.71	0.38	0.20	0.36
C3, wt%	0.83	0.70	0.18	0.57
C4, wt%	0.67	0.43	0.28	0.37
C5–C6, wt%	0.54	0.36	0.17	0.23
C6+, wt%	0.01	0.01	0.01	0.10
Unknown, wt%	0.10	0.06	0.10	0.65
H_2_, wt%	0.36	0.26	0.07	0.52

**Table 6:** Gaseous products obtained from catalytic tests performed in CO_2_. The quantity of gases was calculated from the total amount of products obtained in wt%.

	PP-MgO-CO2	PE-MgO-CO2	PP-CaO-CO2	PE-CaO-CO2
C1, wt%	0.34	0.58	0.09	0.56
C2, wt%	0.72	0.33	0.17	1.16
Ethylene, wt%	0.03	0.06	0.01	0.15
Propylene, wt%	0.74	0.19	0.21	0.81
C3, wt%	0.62	0.39	0.15	1.22
C4, wt%	0.65	0.26	0.27	0.81
C5-C6, wt%	0.36	0.15	0.21	0.39
C6+, wt%	0.04	0.01	0.03	0.12
Unknown, wt%	0.13	0.06	0.04	0.75
H_2_, wt%	0.13	0.13	0.07	1.25
CO, wt%	0.17	0.51	0.04	0.44

**Table 7:** Refractive Index and densities for the liquid products.

	Refractive Index (20 °C)	Density (15 °C), kg m^−3^
PP-MgO-N2	1.5238	933.10
PE-MgO-N2	1.5265	934.54
PP-CaO-N2	1.5238	934.98
PE-CaO-N2	1.5174 (50 °C)	947.33 (50 °C)
	Refractive Index (20 °C)	Density (15 °C), kg m^−3^
PP-MgO-CO2	1.5254	938.89
PE-MgO-CO2	1.5263	942.40
PP-CaO-CO2	1.5251	939.62
PE-CaO-CO2	1.5181 (50 °C)	949.12 (50 °C)

**Table 8:** Other published results compared with the results in this work.

Catalyst	Catalyst wt%	Feed	T °C	Liquid wt%	Gas wt%	Solid wt%	Ref.
ZSM-5	10	PE, PP, PS, PET, PVC	450	56.9	40.4	3.2	[12,32]
ZSM-5	10	PE, PP, PS, PET, PVC	440	39.8	58.4	1.8	[12,32]
Red Mud	10	PE, PP, PS, PET, PVC	500	57.0	41.3	1.7	[12,32]
BAC/MgO ^1^	66.6	PE	500	81.0	15.1	3.9	[13]
CaO	20	PP	420	84.7	1.2	14.1	This work
MgO	20	PE	420	81.9	6.9	2.1	This work
p-toluene sulfonic acid ^2^	3	Biomass	120–180	96.2	--	--	[8]
Mo/C ^3^	0.5	Coal + PE	420	90	8	2	[9]
No catalyst	--	Waste plastic	400	49	30	21	[34]
CoMo/Al_2_O_3_	5	Waste polyolefins	400–500	81	19	--	[35]
HZSM-5	3	PE	430	95–99	--	--	[36]
No catalyst	--	PE, PV, PET	440	85	--	--	[37]

^1^ (BAC/MgO) Biomass derived activated carbon/MgO. ^2^ Thermochemical liquefaction using acids and biomass. ^3^ The work using Mo/C was carried out using 1 wt% of Mo impregnated over coal.

## Data Availability

The data presented in this study are available on request from the corresponding author. The data are not publicly available due to privacy concerns.

## Supplementary Materials

The following are available online at https://www.mdpi.com/article/10.3390/ma15030844/s1, Figure S1: SIMDIS pattern of all the products from the tests carried out in N_2_, Figure S2: SIMDIS pattern of all the products from the tests carried out in CO_2_, Figure S3: TGA analyses in N_2_, Figure S4: TGA analyses in O_2_, Figure S5: ATR-FTIR for liquid and solid products using waste propylene plastic in N_2_ atmosphere, Figure S6: ATR-FTIR for liquid and solid products using waste propylene plastic in CO_2_ atmosphere, Figure S7: Mass balance for the obtained products using the waste plastic under N_2_ or CO_2_, Figure S8: SIMDIS for the liquid products from the waste propylene plastic obtained under N_2_ or CO_2_, Table S1: Results from the analysis of physisorption (Samples were degassed in Autosorb iQ at 110 ° C for 24.3 h
