# Supplementary material for: Direct Polypropylene and Polyethylene Liquefaction in CO2 and N2 Atmospheres Using MgO Light and CaO as Catalysts
Source: Materials (Basel). 2022 Jan 22;15(3):844. doi: 10.3390/ma15030844 (PMC8836839; doi:10.3390/ma15030844)
Supplement: Supplementary file 1 [file materials-15-00844-s001.zip › materials-1540623-supplementary.pdf]

materials

Supplementary Materials

Direct Polypropylene and Polyethylene Liquefaction in CO_2 and N_2 Atmospheres Using MgO Light and CaO as Catalysts

José Miguel Hidalgo Herrador *, Martyna Murat, Zdeněk Tišler, Jakub Frątczak and Héctor de Paz Carmona

ORLEN UniCRE a.s., Revoluční 1521/84, 400 01 Usti nad Labem, Czech Republic;
martyna.m.murat@gmail.com (M.M.); zdenek.tisler@orlenunicre.cz (Z.T.); jakub.fratczak@orlenunicre.cz (J.F.); hector.carmona@orlenunicre.cz (H.d.P.C.)
* Correspondence: jose.hidalgo@orlenunicre.cz

As supplementary information, simullated distillation (SIMDIS), TGA, ATR and mass balance analysis results are exposed in Figures S1–S8 for an additional test carried out for a polypropylene (PP) waste (a mixture of polypropylene waste from cars recycling). These results are included here because it was not the main purpose of the original manuscript but we consider this information really useful for researchers and people interested in this type of reactions applied for real plastics. In addition, some more analyses would be needed such as the halogenes analysis, metals analysis (high ash content according to TGA results).

Complete patterns of SIMDIS were included (for all the tests results) in Figures S1 and S2.

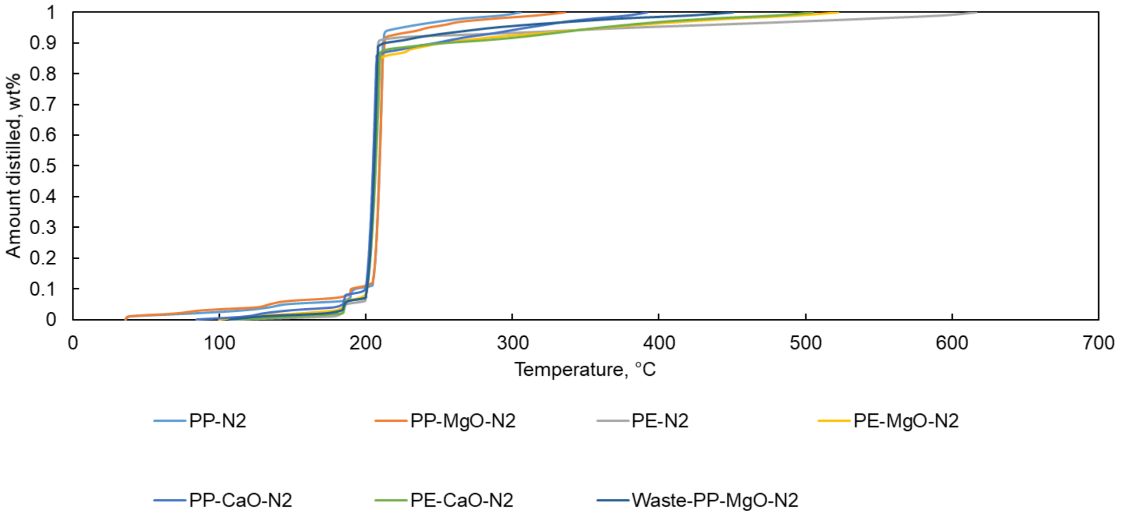

Figure S1. SIMDIS pattern of all the products from the tests carried out in N_2.

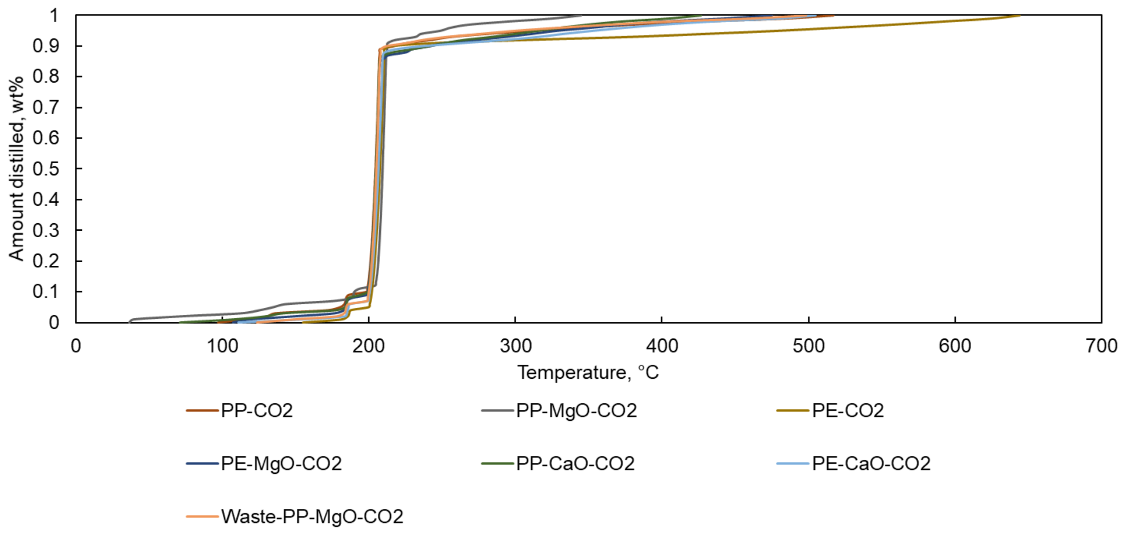

Figure S2. SIMDIS pattern of all the products from the tests carried out in CO_2.

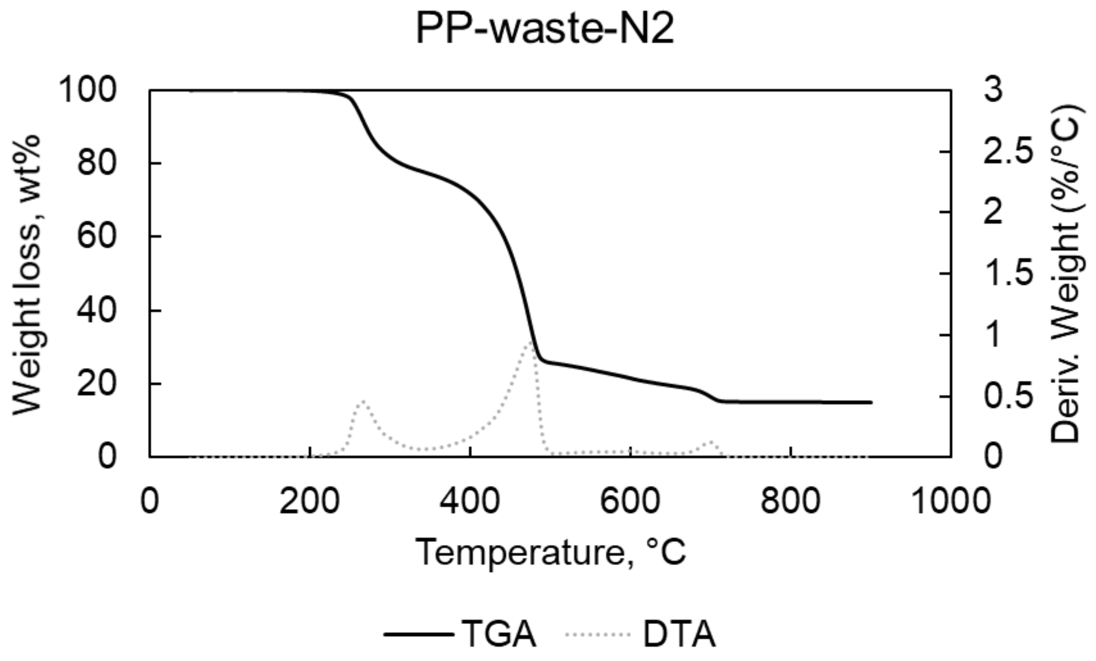

Figure S3. TGA analyses in N_2.

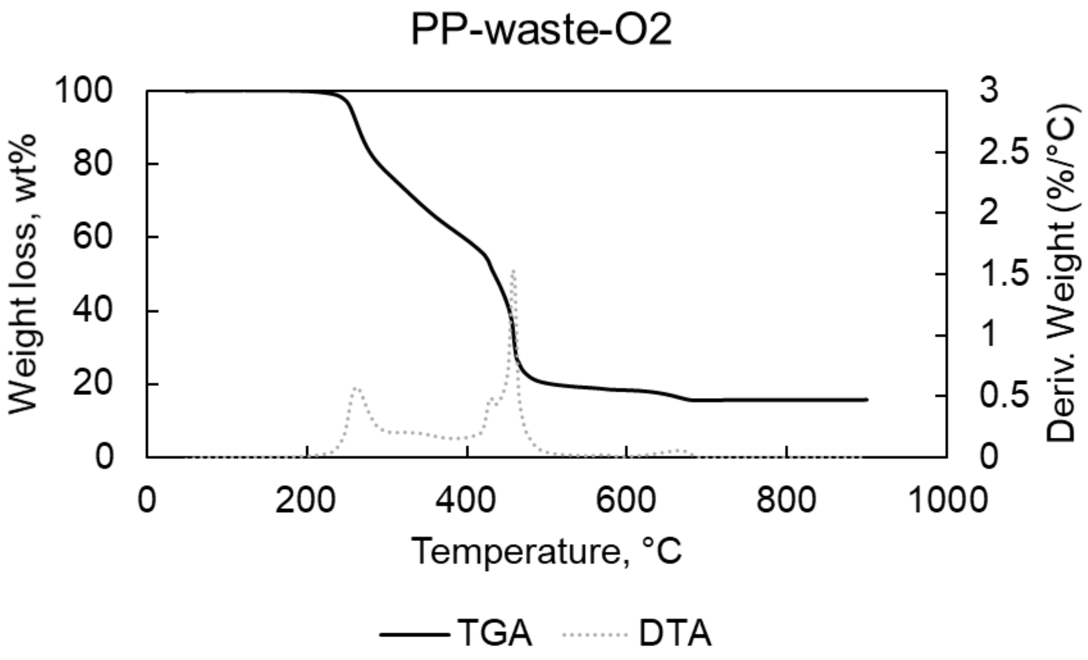

Figure S4. TGA analyses in O_2.

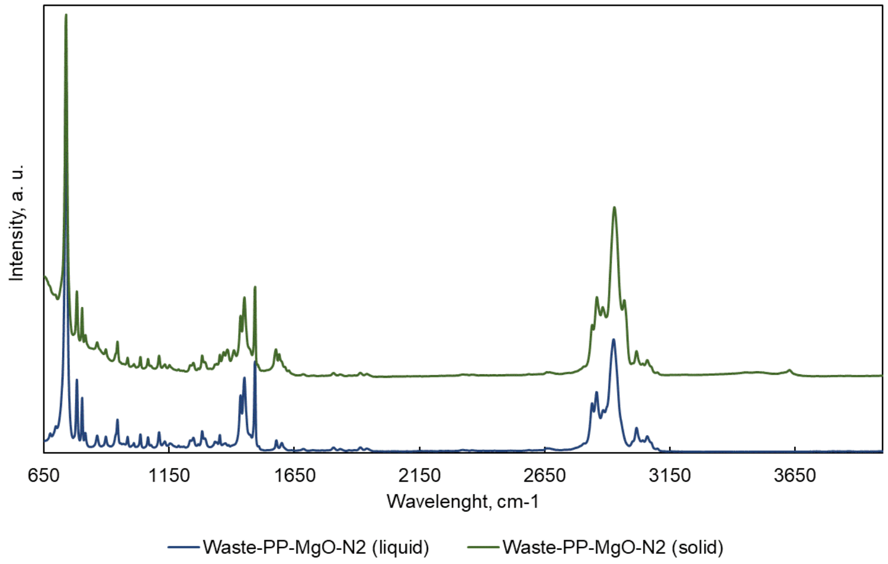

Figure S5. ATR-FTIR for liquid and solid products using waste propylene plastic in N_2 atmosphere.

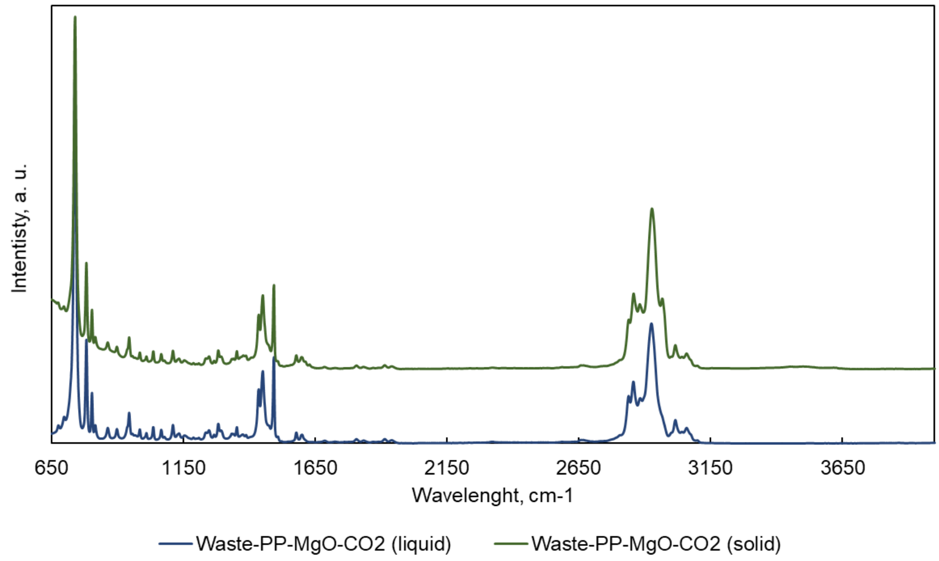

Figure S6. ATR-FTIR for liquid and solid products using waste propylene plastic in CO_2 atmosphere.

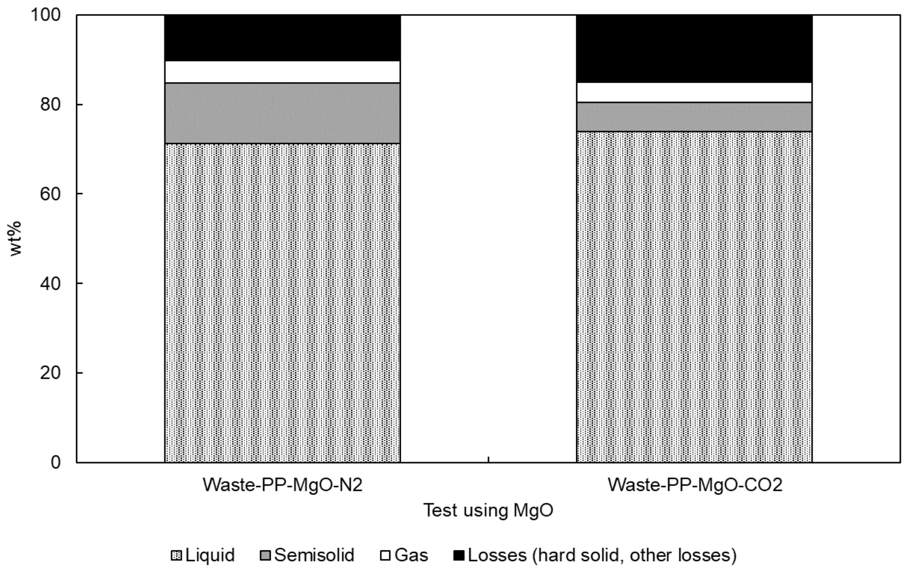

Figure S7. Mass balance for the obtained products using the waste plastic under N_2 or CO_2.

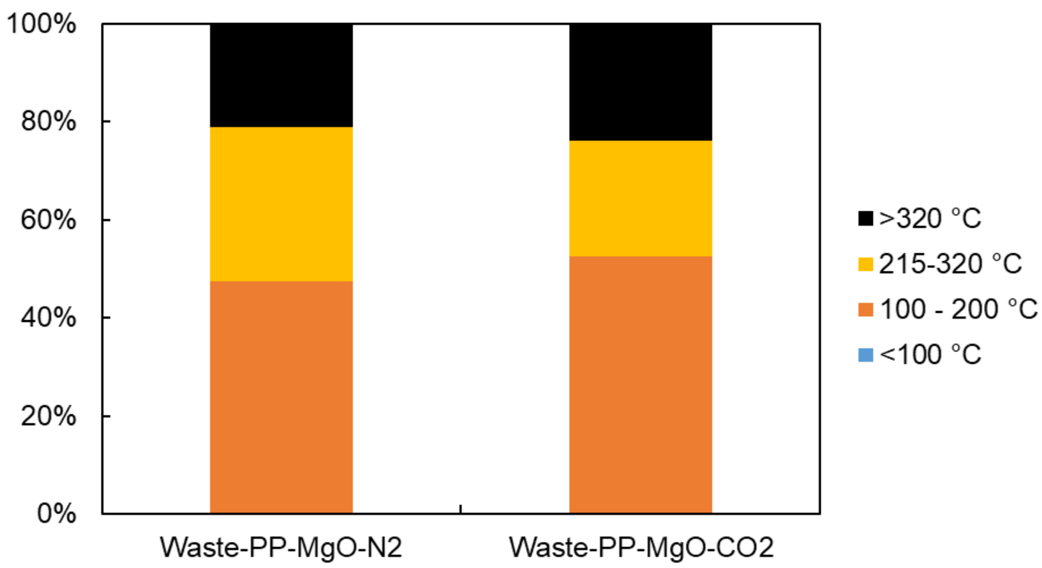

Figure S8. SIMDIS for the liquid products from the waste propylene plastic obtained under N_2 or CO_2.

Table S1. Results from the analysis of physisorption (Samples were degassed in Autosorb iQ at 110 °C for 24.3 h.

Parameter	MgO Light	CaO
**Isotherm type**	IV	IV
**Hysteresis**	H3	H3
**Total pore volume [cm³ / g]**	**0.236**	**0.054**
**BET specific surface area [m² / g]**	**36.1**	**10.0**
Range p / p0 for SBET	0.10–0.25	0.15–0.30
Number of points	4	4
Constant C	90	23
Correlation coefficient, r	0.999991	0.999982
**V-t analysis**		
Micropore volume [cm³ / g]	0.000	0.000
Outer surface [m² / g]	36.1	10.0
**NLDFT analysis**		
Specific surface area [m² / g]	40.3	8.2
Total pore volume [cm³ / g]	0.189	0.027
Micropore volume [cm³ / g]	0.000	0.000
Mesopore volume [cm³ / g]	0.166	0.019
NLDFT model match [%]	97.44	98.01
NLDFT model	2	2
**BJH analysis**		
Specific surface area [m² / g]	41.4	7.4
Total pore volume [cm³ / g]	0.237	0.053
Mesopore volume [cm³ / g]	0.188	0.019
